# In situ pulmonary artery thrombosis after proton beam therapy

**DOI:** 10.1007/s11604-026-02022-5

**Published:** 2026-06-15

**Authors:** Masaaki Goto, Toshiki Ishida, Hideyuki Sakurai

**Affiliations:** Department of Radiation Oncology & Proton Medical Research Center, Institute of Medicine, University of Tsukuba, Amakubo 2-1-1, Tsukuba, Ibaraki 305-8576 Japan

In situ pulmonary artery thrombosis (PAT) is an underrecognized entity and one of the mimickers of pulmonary embolism (PE) [1]. Although PAT presents on imaging as a filling defect within the pulmonary artery, the thrombus is thought to arise de novo from the pulmonary arterial wall in the absence of deep vein thrombosis (DVT) [2]. Radiation therapy has been reported as one of its possible causes (3); however, only a limited number of cases have been described.

**Fig. 1:**
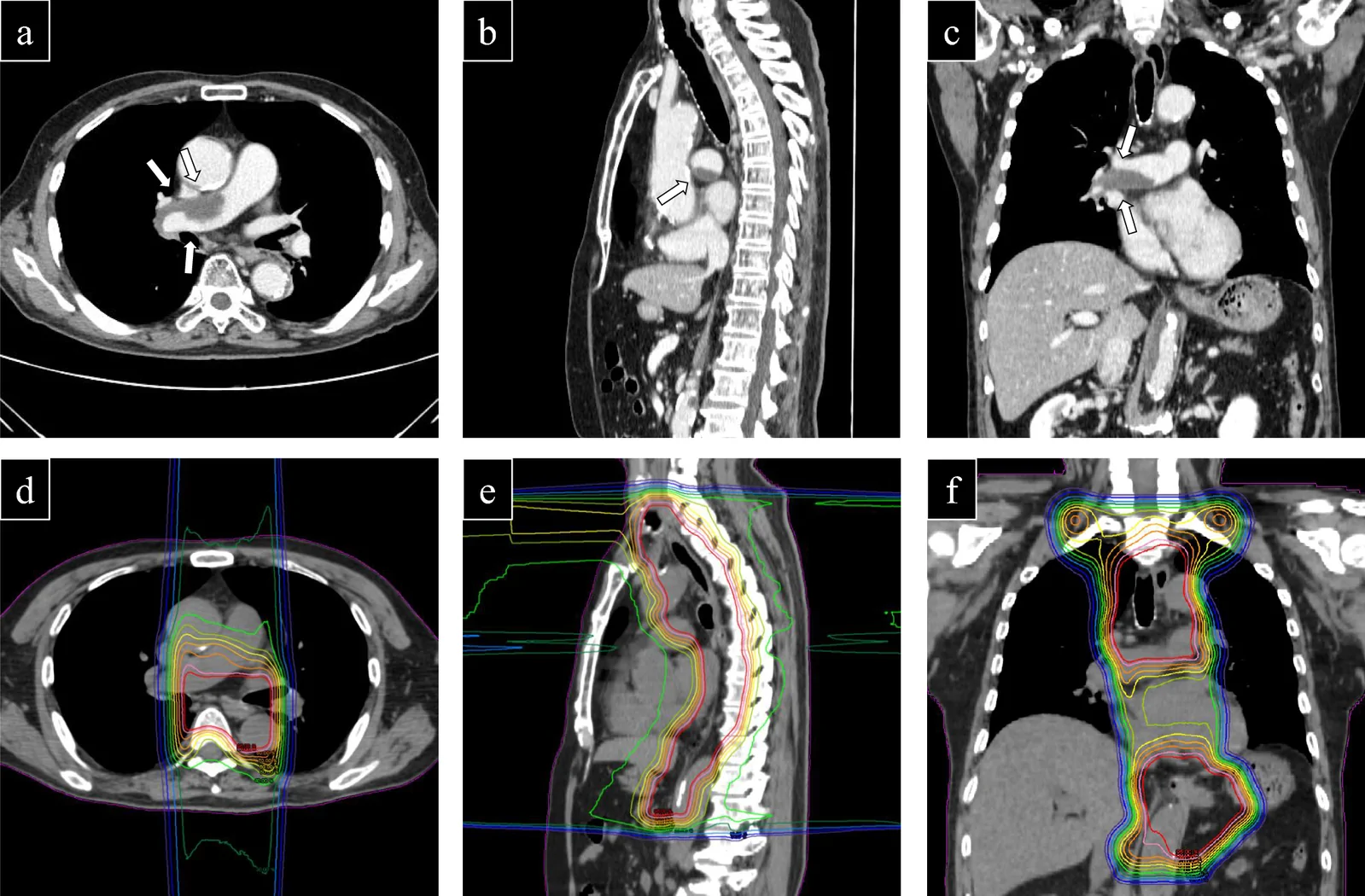
In situ pulmonary artery thrombosis after proton beam therapy. **a**-**c** Contrast-enhanced CT images obtained 32 months after proton beam therapy (PBT) for esophageal cancer. Axial (**a**), sagittal (**b**), and coronal (**c**) views demonstrate a large, eccentric filling defect within the right pulmonary artery without contrast enhancement (arrows). **d**-**f** Corresponding CT images overlaid with PBT dose distribution (**d**: axial, **e**: sagittal, **f**: coronal). The isodose lines are shown in rainbow colors, with the red line representing the 95% prescribed dose. The filling defect in the right pulmonary artery corresponds to the mediastinal irradiation field

An 80-year-old man with a history of concurrent chemoradiotherapy using proton beam therapy (66 Gy in 33 fractions) for middle thoracic esophageal cancer (cT1bN0M0, Stage I) remained recurrence-free for 32 months after treatment. On routine follow-up contrast-enhanced CT examination, a relatively large eccentric filling defect was incidentally identified in the right pulmonary artery (Fig. 1a–c). Retrospective review confirmed that no filling defect had been observed at the corresponding site on pre-treatment or previous follow-up CT images. In addition, the lesion showed no contrast enhancement, making tumor embolism less likely. The patient was asymptomatic, and his vital signs were stable. The patient had no history of venous thromboembolism, recent surgery, or prolonged immobilization. The D-dimer level was elevated at 3.4 μg/mL. Lower-extremity ultrasonography showed no evidence of DVT. Although the lesion was initially interpreted as PE, the absence of DVT, the lack of apparent clinical thrombotic risk factors, and its location within the irradiation field of proton beam therapy (Fig. 1d–f), where the involved pulmonary artery received a maximum dose of 66 Gy, supported the final diagnosis of in situ PAT rather than PE.

This case had two atypical findings compared with previous reports. First, there was a discrepancy between the high-dose region and the site of thrombus formation. Because the relationship between dose distribution and thrombus localization has not been fully established, further accumulation of cases is needed. Second, no obvious radiation-induced pulmonary fibrosis was observed, although pulmonary fibrosis has been described in previous reports. The absence of pulmonary fibrosis may partly reflect the dose distribution of proton beam therapy, which can reduce the lung dose while delivering a steep dose increase at depth.

To our knowledge, this is the first reported case of in situ PAT after proton beam therapy, highlighting that in situ PAT should be considered in the differential diagnosis of pulmonary arterial filling defects in patients with a history of mediastinal irradiation involving the pulmonary artery, including proton beam therapy.
